# Comparison of Nutritional and Meat Quality Characteristics between Two Primal Cuts from Aceh Cattle in Aceh Province, Indonesia

**DOI:** 10.1155/2021/8381849

**Published:** 2021-08-17

**Authors:** Hamny Sofyan, Aryani Sismin Satyaningtijas, Cece Sumantri, Etih Sudarnika, Srihadi Agungpriyono

**Affiliations:** ^1^Laboratory of Anatomy, Faculty of Veterinary Medicine, Universitas Syiah Kuala, Banda Aceh 23111, Indonesia; ^2^Department of Anatomy, Physiology, and Pharmacology, Faculty of Veterinary Medicine, IPB University, Bogor 16680, Indonesia; ^3^Department of Animal Production and Technology, Faculty of Animal Science, IPB University, Bogor 16680, Indonesia; ^4^Department of Animal Infectious Diseases and Veterinary Public Health, Faculty of Veterinary Medicine, IPB University, Bogor 16680, Indonesia

## Abstract

The Aceh cattle are local Indonesian beef cattle that are farmed in Aceh Province. This type of cattle is one of the sources of meat for the Aceh people. This study aims to analyze the quality of two primal cuts (longissimus lumborum and semitendinosus muscle) from Aceh cattle based on the muscle microstructure characteristics and MSTN gene expression. This study used a sample of longissimus lumborum and semitendinosus muscles from 18 adult male Aceh cattle with the age of 2–2.5 years and a BCS of 3.24. Muscle samples were obtained shortly after the cattle were slaughtered in slaughterhouses in Banda Aceh and Aceh Besar districts. Muscle microstructure analysis was performed using the HE, Masson's trichrome, and immunohistochemistry staining methods, while the MSTN gene expression analysis was performed using the qPCR method. The analysis of the physical quality of meat includes pH, meat color, fat color, cooking loss, water holding capacity, and WBSF value. The results showed that the area of LL muscle fibers was smaller than that of ST with relatively the same diameter. Both muscles were dominated by fast fibers with a percentage of 82.37% (LL muscle) and 91.80% (ST muscle). The area and composition of the type of muscle fibers are the main factors that influence the tenderness of Aceh beef. A higher distribution of collagen was found in ST muscles than in LL muscles. MSTN gene expression in both muscle types was relatively the same. Aceh cattle have large muscle fibers and are dominated by fast fibers with a high percentage, resulting in a low level of the tenderness of Aceh beef. However, the level of tenderness of Aceh beef is still in accordance with the cooking preparation of original and favorite cuisine of Aceh people.

## 1. Introduction

The quality of meat is an important factor as a reference for consumers to consume meat, but the quality of meat is not yet a major limiting factor for consumers to buy meat in Indonesia. However, efforts to provide good quality beef for consumers are important. Many factors affect the quality of meat, including genetics, environment of maintenance [1], feed [2], stress before slaughter [3], physical activity [4], weight and age at slaughter [5], and gender [6]. All of these factors can affect the quality of the meat through the image of the muscle microstructure. Muscle microstructure can show the quality of the meat. Muscle microstructure features that are often analyzed about meat quality include the number and diameter of muscle fibers, sarcomere length, collagen content, and muscle fiber type composition [6]. The muscle microstructure correlates with the proteins involved in the formation of muscle cells (myofibrils). One of these proteins is myostatin. Myostatin is a member of transforming growth factor-*β* (TGF-*β*), which is encoded by the myostatin gene (MSTN), which functions as the main regulator of the muscle-building process (myogenesis) and skeletal muscle growth [7]. Overexpression of myostatin causes a decrease in muscle mass, muscle fiber area, and muscle protein [8].

Longissimus lumborum and semitendinosus muscle are two types of muscle constructing meat primal cuts preferred by the consumer in Aceh Province, Indonesia [9]. Meat cuts from longissimus lumborum muscle had a higher price than commercial meat cuts from semitendinosus muscle. Such price difference is caused by the meat tenderness difference between the cuts. Meat tenderness is affected by its constructed muscle composition and structure [10]. Longissimus lumborum and semitendinosus muscles have different compositions and structures because their anatomical locations and physiological functions are different [10]. Muscle growth becomes important and receives attention from the breeder and meat industry.

The muscle growth characteristics can increase the economic value of livestock, in addition to feeding efficiency [11]. Previous studies have also shown that the composition of muscle fiber type is one of the muscle growth characteristics variables which affects the quality of meat, especially concerning meat palatability, including affecting the taste (umami and richness) [12], meat color, pH, water binding capacity, tenderness, and nutritional value of meat [13–15], components of connective tissue and intramuscular fat [15, 16], and amino acid composition [17]. Wegner et al. [18] state that for cattle fattening and cow breeding businesses, a good understanding of muscle characteristics is important to produce meat with maximum quantity and quality. Muscle mass can be maximized through the number and size of muscle fibers, as well as muscle fiber transformation. A comprehensive analysis of beef quality based on muscle microstructural characteristics has been carried out in Europe, Australia, Japan, America, and other developed countries, but the same study has never been carried out on local Indonesian beef cattle.

The quality of meat originating from smallholder farms has never been studied before, so this study is important to do as a baseline for the preparation of beef quality standards in Indonesia, such as the USDA (United States Department of Agriculture), MSA (Meat Standards Australia), and JMGA (Japan Meat Grading Association) as a reference for beef quality standards in America, Australia, and Japan [19, 20]. The standard of beef quality in Indonesia currently refers to the Indonesian National Standard (SNI) 3932–2008 concerning Carcass and Beef Quality [21]; however, it does not cover all indicators of meat quality due to limited data.

The quality of Indonesian local beef is still below the quality of imported beef [22, 23] because it is still raised traditionally. However, meat markets in advanced countries have added meat quality standards related to animal welfare and environmental sustainability aspect in the past several years [24]. Therefore, an investigation is necessary for Indonesian traditional local cattle breeding to know the meat quality produced as a baseline for future meat quality improvement programs. Other studies on Indonesian local beef cattle quality (Bali cattle and Ongole grade/PO cattle) showed the relationship between meat quality and muscle microstructure [22, 23]. However, these two studies have not examined the composition of muscle fiber type which is also an important factor for meat quality. The composition of muscle fiber types can be engineered to produce more optimal meat production with preferred tenderness through genetic manipulation or breeding management modification. Therefore, this study aims to analyze the quality of Aceh beef originating from smallholder farms based on muscle microstructure and MSTN gene expression. The results of this study can be used to improve the quality standards of Indonesian local beef and become a reference for government policymaking to improve the quality of beef in Indonesia.

## 2. Materials and Methods

### 2.1. Ethical Approval

The study has received ethical approval from the IPB Ethics Committee of 2018 Number: 101–2018 IPB.

### 2.2. Sample Collection

The study used meat samples from 18 adult male Aceh cows aged ± 2–2.5 years and had an average body condition score (BCS) of 3.24. The number of Aceh beef cattle used was relatively few because of the low participation of Aceh beef cattle owners in this study and the low number of Aceh beef cattle cut in a slaughterhouse during the current research period. This was assumed as a result of the recent decrease of Aceh beef cattle population. Meat samples were taken at the Keudah slaughterhouses in Banda Aceh and Lambaro, Aceh Besar District, and the traditional slaughterhouses in Bayu Village and Lhong Cut Village, Banda Aceh, Aceh Province, Indonesia. The age range of Aceh cows is quite wide due to the absence of a recording system by the breeders so that the determination of the age of the cows is based on teeth. The Aceh cattle used in the study did not experience a stunning process before slaughter and were not castrated, and the resulting carcass did not undergo chilling at 4°C for 24 hours. The meat samples came from the longissimus lumborum (LL) and semitendinosus (ST) muscles. The determination of LL and ST muscles as samples is based on the following. (i) These two muscles are the constituent of meat that is often purchased by consumers. (ii) The two muscles are different in their constituent contractile and metabolic components which are known from the anatomy and function of the two muscles. LL muscle represents passive muscle and is fast oxidoglycolytic, whereas ST muscle represents active muscle and is fast glycolytic. (iii) Both muscle types are often used as muscle samples in meat quality studies.

Each meat sample is taken immediately after the livestock is slaughtered by 500 g of each type of muscle taken and divided into four parts. They are used for muscle microstructure analysis (meat is put into 10% neutral buffered formalin (NBF) fixation solution), proximate analysis (meat is put into plastic), physical quality analysis (meat is put in a plastic container and in a vacuum), and MSTN gene expression analysis (the meat is put into an Eppendorf tube that has been filled with RNAlater stabilization solution) (RiboSaver, GeneAll, Korea). Meat samples for proximate analysis, physical quality, and MSTN gene expression were immediately put into a cool box containing ice gel, which was then taken to the laboratory for storage at −20°C and −80°C until the analysis was carried out.

### 2.3. Muscle Microstructure Analysis

This analysis begins with the manufacture of histological preparations. Muscle samples were fixed with 10% NBF, dehydrated with 70%, 80%, 90%, 95%, and absolute alcohol solutions, cleared with xylol, paraffinized with liquid paraffin, and embedded to block paraffin. Paraffin blocks were cut using a microtome with a thickness of 5 *μ*m.

Muscle microstructure analysis includes measuring the diameter and area of muscle fibers, observing the distribution of collagen, and calculating the percentage composition of muscle fiber types. Muscle microstructure analysis was performed with several types of staining. Hematoxylin-eosin (HE) staining was used to measure the diameter and area of muscle fibers. Masson's trichrome (MT) staining was used for the observation of collagen distribution. Immunohistochemical (IHC) staining with anti-slow skeletal myosin heavy chain (MHC) I antibody (dilution 1 : 100, cat no. A083, Abcam, Cambridge, UK) was used to identify slow fiber muscle type (type I) and anti-fast MHC II antibody (dilution 1 : 200, cat no.ab75370, Abcam, Cambridge, UK) for the identification of fast fiber muscle types (type II). The IHC staining procedure followed the instructions on the IHC kit used, namely, mouse- and rabbit-specific HRP/DAB (ABC) detection immunohistochemistry kit (cat no. Ab64264, Abcam, Cambridge, UK) with a modification in the form of giving 0.1% triton-x as much as 10 *μ*l along with the administration of primary antibodies. The calculation of the percentage of muscle fiber types followed the procedure of Hwang et al. [6].

### 2.4. Proximate Analysis and Physical Quality of Meat

The proximate analysis includes the percentage of protein content using the Kjedahl method, fat content using the Soxhletation method, moisture content, and ash content using the Gravimetric method. The analysis of the physical quality of meat included meat pH after 24 hours of cutting (pH_24_), meat color, fat color, intramuscular fat, meat tenderness, cooking loss, and water holding capacity. pH_24_ measurements were carried out using a pH meter (HI 99163; Hanna, Woonsocket, USA) by inserting a probe into the meat. The assessment of meat and the fat color is carried out by visually observing the surface color of meat and fat and matching it with the standard meat and fat color according to the applicable standards in Indonesia, namely, SNI 3932 : 2008 (scale 1–9). The marbling assessment is carried out by looking at the intensity of marbling on the surface of the meat and matching it with the marbling standards according to SNI 3932 : 2008 (scale 1–12).

Meat tenderness is obtained from the Warner-Bratzler shear force (WBSF) value. The meat sample of 200 g (±10 cm thickness) is inserted by a bimetal thermometer until it penetrates the inside of the meat, then it is put into a pot of water until the meat is completely submerged, and then the meat is boiled until the thermometer shows the number 81°C. The meat was molded using a corer, and the meat was measured to determine its breaking strength (kg cm^−2^) using the Warner-Bratzler tool [6].

Cooking losses are the difference between the weight of meat before and after cooking and are expressed as a percentage (%). About 100 g of meat is plugged in a bimetallic thermometer until it penetrates the inside of the meat and is put into water and boiled. Meat samples must be immersed in boiling water until the temperature in the meat shows the number 81°C and then removed. The meat is cooled for 60 minutes and then weighed. Water binding is carried out by the press method. A 0.3-gram sample of meat was placed between two filter papers and then given the pressure of 35 kg for five minutes. The area covered by the meat sample and the area of the surrounding wetlands are marked and measured with a planimeter. The wet area is obtained by calculating the difference between the area covered by meat from the total area which includes the wet area on the filter paper.

### 2.5. RNA Extraction from Muscle Samples and Quantitative Polymerase Chain Reaction (qPCR)

The analysis of MSTN gene expression was done using the quantitative polymerase chain reaction (qPCR) method. The primer design for the MSTN gene and *β*-actin housekeeping gene was done using Mega 6 software with accession numbers NM_001001525.3 and XM_019987862.1, respectively. The specific primer sequences that have been designed and used in the study are presented in Table 1.

The meat samples analyzed were from 8 LL muscles and 8 ST muscles. The total mRNA was isolated using the RNeasy Fibrous Tissue Mini Kit (paint. No. 74704, Qiagen, Germany). The total RNA isolation procedure followed the procedure from the kit used with a slight modification in the form of increasing the amount of proteinase K given as much as 20 *μ*l. Each meat sample was taken as much as 30 mg and separated from RNAlater and then homogenized with the help of liquid nitrogen (−196°C) using a micropestle. The next stage follows the procedure of the kit used. The concentration and purity of mRNA were measured using a nanodrop (Thermo Scientific, US) with an absorbance of 260/280 nm. Pure RNA is stored at −80°C until the next step is carried out. The synthesis of complementary deoxyribonucleic acid (cDNA) was carried out using 100 ng of total RNA that had been purified using ReverTra Ace qPCR RT Master Mix with gDNA Remover (FSQ-301, Toyobo, Japan). The obtained cDNA was stored at −20°C. The analysis of gene expression was done using the qPCR tool (qTower, AnalyticJena, Germany). The cDNA amplification and quantification process used Thunderbird SYBR qPCR Mix (Toyobo, Japan). The concentration of cDNA used was 100 ng/*μ*l. The PCR reaction used was 11 *μ*l, and each PCR reaction consisted of 2 × SYBR Green Master Mix of 5 *μ*L, forward and reverse primer (previously these two primers had been mixed) of 1 *μ*L, cDNA template of 2 *μ*L, and RNase-free water of 3 ml. The PCR conditions used were predenaturation at 95°C for 1 minute, denaturation at 95°C for 15 seconds, annealing at 55°C for 1 minute, and extension at 72°C for 30 seconds with many cycles of 40 times.

### 2.6. Data Analysis

The measurement of fiber area and diameter as well as the calculation of muscle type composition was done using ImageJ software. Collagen distribution was analyzed quantitatively. All data (except collagen distribution) are presented as means and standard deviations. Differences between muscle groups were analyzed using the Independent-samples T-test. The quantification of the relative expression of the MSTN gene was calculated based on the relative number of MSTN and housekeeping genes (*β*-actin) as internal control genes based on the cycle threshold (Ct) value. The value of Ct is calculated from the average of three measurements. The comparative quantification of Ct values used the ΔΔCT method [25].

## 3. Results

### 3.1. Muscle Microstructure

**Figure 1:**
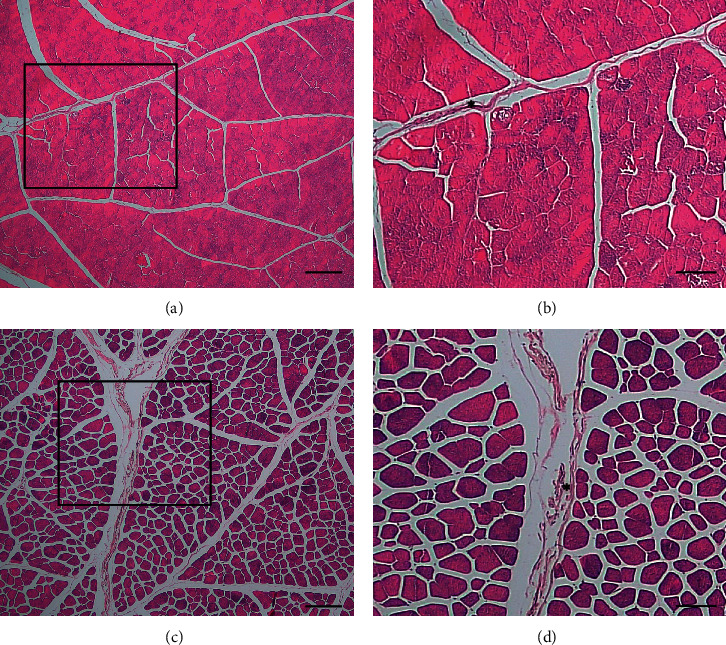
ST muscles are composed of muscle fibers that are larger and contain a lot of collagen (^*∗*^) connective tissue. Hematoxylin-eosin stain. Bars 200 *μ*m (a, c) and 100 *μ*m (b, d).

**Figure 2:**
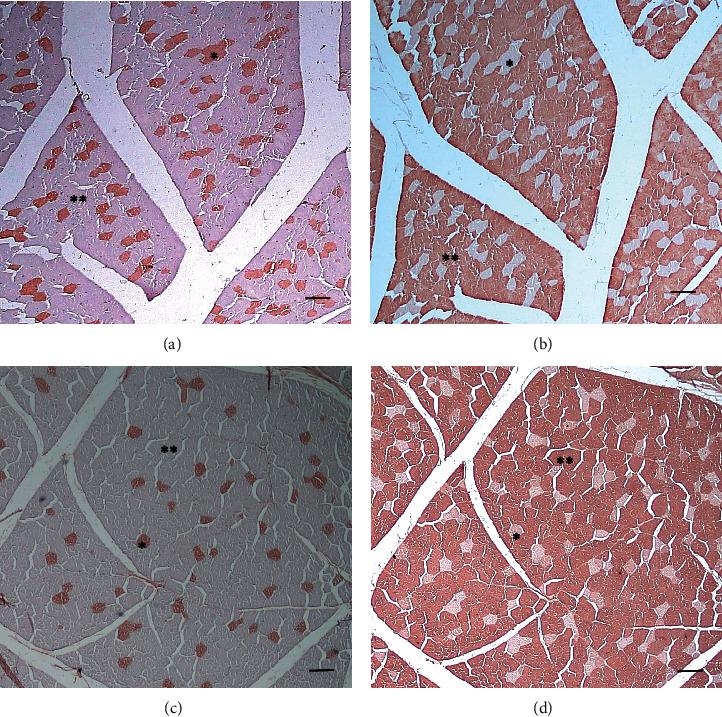
Types of longissimus lumborum (LL) (a, b) and semitendinosus (ST) (c, d) muscle fibers in Aceh cattle. The two types of muscle consist of type I (^*∗*^) and type II (^*∗∗*^) muscle fibers. Type II fibers predominate in both muscle types. Immunohistochemical staining. Bars 100 *μ*m (a–d).

The longissimus lumborum (LL) muscle has a smaller fiber area than the semitendinosus (ST) muscle (*p* < 0.05), while the diameter between the two muscles is not different (*p* > 0.05) (Table 2 and Figure 1). The area of LL muscle fibers was 3409.66 ± 1072.27 *μ*m^2^ with a diameter of 45.58 ± 7.95 *μ*m, while the area of the ST muscle fibers was 3566.70 ± 1077.20 *μ*m^2^ with a diameter of 46.80 ± 7.07 *μ*m. The percentage of types I and II in the two muscle types did not differ significantly (*p* > 0.05). LL muscle has type I fibers with a percentage of 17.63 ± 13.96% and type II as much as 82.37 ± 13.96%. ST muscle has type I fibers with a percentage of 8.20 ± 3.20% and types II as much as 91.80 ± 3.20% (Table 2 and Figure 2).

**Figure 3:**
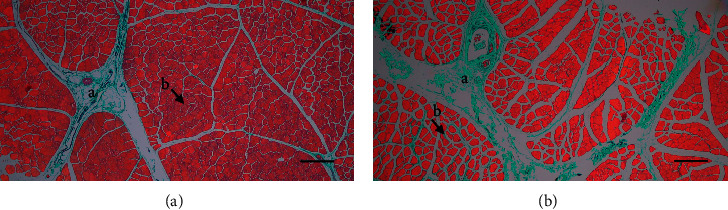
Distribution of collagen tissue in the longissimus lumborum (LL) (a) and semitendinosus (ST) (b) muscles in Aceh cattle. Collagen tissue (green in color) is more common in the ST muscle, which is distributed more in the perimysium layer (a) than in the endomysium (b). Masson's trichrome staining. Bars 300 *μ*m (a, b).

Table 3 and Figure 3 show the distribution of collagen connective tissue in LL and ST muscles in Aceh cattle. Collagen distribution observations were carried out by Masson's trichrome staining and positive reactions to collagen were marked by green in the collagen-containing tissue. The observation showed that collagen was found in both the endomysium and perimysium muscle layers of LL and ST with different thicknesses. The collagen tissue in the perimysium layer tends to have a higher thickness level (+to +++) compared to the endomysium layer with a thinner thickness level (+). The collagen content in the perimysium layer of the ST muscle is more than that of the LL muscle, while the collagen in the endomysium layer of the two muscles is relatively the same. In this study, the distribution of collagen in the epimysium muscle layer was not observed.

### 3.2. Proximate Analysis and Physical Quality of Meat

The results of the proximate analysis of Aceh beef are presented in Table 4. The percentage of protein content, fat content, water content, and ash content in the two types of muscle did not differ significantly (*p* > 0.05). The percentages of protein, fat, water, and an ash content of LL muscle meat were 20.90 ± 1.27%, 2.69 ± 2.00%, 73.89 ± 2.26%, and 1.83 ± 0.54%, while those of the protein, fat, water, and an ash content of ST muscle meat were, respectively, 21.61 ± 1.33%, 2.16 ± 1.57%, 73.25 ± 3.57%, and 2.06 ± 0.43%, respectively.

The results of the physical quality analysis of Aceh beef are presented in Table 5. The pH_24_, meat color, and intramuscular fat were significantly different (*p* < 0.05) between the two types of muscle, while for the other variables there was no significant difference (*p* > 0.05). The pH_24_ values of LL and ST muscles were 5.38 ± 0.10 and 5.59 ± 0.13, respectively. LL muscle has an average color score of 3.00 ± 0.00, while ST muscle has an average color score of 2.36 ± 0.74. In the LL muscle, there was also intramuscular fat (marbling) with a mean score of 1.29 ± 0.47, while in the ST muscle there was no intramuscular fat (score 0.00 ± 0.00). The WBSF values, the percentage of cooking loss, and the water holding capacity between LL and ST muscles did not differ significantly (*p* > 0.05). The WBSF values for LL and ST muscles were 7.59 ± 1.36 kg cm^−2^ and 7.69 ± 1.26 kg cm^−2^, respectively. The percentages of LL muscle cooking loss were 47.96 ± 3.34% and 45.16 ± 5.01% in ST muscles. The percentage of water binding capacity for LL and ST muscles was 29.86 ± 2.80% and 29.96 ± 1.50%, respectively.

### 3.3. MSTN Gene Expression

**Figure 4:**
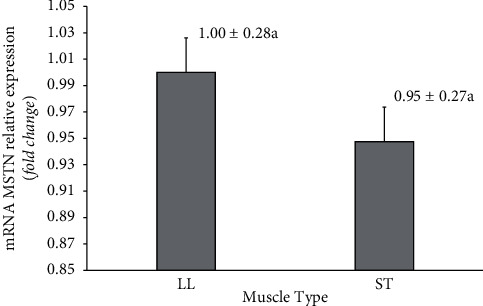
Relative expression of MSTN mRNA in the longissimus lumborum (LL) and semitendinosus (ST) muscles of Aceh cows. Data are presented as means and standard deviations. MSTN mRNA expression in the two muscle types was not significantly different (*p*=0.91).

The relative mRNA expression of the MSTN gene did not differ significantly (*p* > 0.05) in LL and ST muscles. The relative expression of mRNA (fold change) in the LL muscle was 1.00 ± 0.28 and that in the ST muscle was 0.95 ± 0.27 (Figure 4).

### 3.4. The Correlation of the Muscle Microstructure and Meat Quality

Muscle microstructure correlates with MSTN gene expression and meat quality, especially pH, meat tenderness, cooking loss, water holding capacity, fat, and water content (Table 6).

In the LL muscle, the area of muscle fibers has a strong correlation with the MSTN mRNA expression (−0.94) and WBSF value (−0.82) and moderately correlates with cooking losses (−0.36), water holding capacity (0.47), fat content (−0.46), and water content (−0.51). The area of LL muscle fibers has a weak correlation with pH24 (−0.09). Type I and II muscle fibers have a strong correlation with MSTN mRNA expression (0.84), pH24 (0.64), water holding capacity (0.97), fat content (0.96), and water content (0.94) but have a weak correlation with values of WBSF (0.16) and cooking losses (0.29). In the ST muscle, the area of muscle fibers had a strong correlation with the MSTN mRNA expression (0.62), cooking loss (−0.73), fat content (−0.46), and water content (−0.51). The type of fiber in the ST muscle has a strong correlation with the MSTN mRNA expression (0.81), pH24 (0.75), WBSF value (0.79), cooking loss (0.61), water holding capacity (0.80), fat content (0.96), and water content (0.94).).

## 4. Discussion

The study results show that muscle type affects the size of the area of muscle fibers. The area of LL muscle fibers is smaller than that of ST. This difference is thought to be caused by different muscle activities. The ST muscle is an active muscle because it is closely related to livestock movement so that it can stimulate the development of muscle fibers by increasing the size of the muscle fibers. Bee [26] states that increased livestock activity can induce hypertrophy or an increase in muscle cell size as a result of increased protein synthesis. The difference in the area of LL and ST muscle fibers is also thought to be influenced by the expression of the MSTN gene as a protein-coding for myostatin that inhibits muscle cell growth. Although the results of the qPCR analysis showed that the MSTN gene expression in both muscle types was the same, it is suspected that LL and ST muscle responses to the presence of myostatin protein were different. The LL muscle appears to be more responsive to the presence of myostatin protein as indicated by a strong correlation value (−0.94) than in the ST muscle with a lower correlation value (0.62). The area of ST muscle fibers in Aceh cattle is relatively large when compared to the area of ST muscle fibers in other local Indonesian cattle such as Bali cattle and PO cattle reported by Safitri et al. [23] but smaller than the size of the muscle fibers in *Bos taurus* cattle as reported by Wegner et al. [18]. Aceh cows are a cross of *Bos indicus* cattle and belong to a small group of cattle, so they tend to have smaller muscle fiber sizes compared to exotic cattle (*Bos taurus*).

Bovine skeletal muscle is composed of several types of muscle fibers, namely, type I, type IIA, and type IIX [16]. Muroya et al. [27] classify muscle fibers into 2 types, namely, slow fiber type (type I) and fast fiber type (type II). The study results showed that both LL and ST muscles in Aceh cattle were dominated by type II muscle fibers (fast fiber) with a high percentage. However, this study did not differentiate between muscle fiber subtypes. This result is supported by a study conducted by Hwang et al. [6] on Hanwoo (local Korean cattle), although the percentage of type IIB fibers is not very high. The longissimus thoracis (LT) and ST muscles in *Bos taurus* (Charolais) cattle were dominated by type IIA and type IIX + *B*, respectively [16]. The high percentage of type II muscle fibers in the two types of muscle in Aceh cattle is thought to be influenced by genetic factors. Aceh cows are the result of crossing *Bos indicus* with *Bos javanicus*, so that the LL and ST muscles of Aceh cows tend to show a higher presence of type II fibers. The composition of the type of muscle fibers is also influenced by gender, age, muscle type, breed, and type of livestock [16]. The percentage of type IIB muscle fibers in Hanwoo cattle is found in the highest quality meat (grade I), while the Aceh beef sample used in this study is not known for its quality because the grading system has not been implemented in the Aceh beef sales process. Lorenzo et al. [28] stated that the composition of muscle fiber types has been shown to contribute to producing meat with the level of tenderness desired by consumers. Meat produced by muscles that contain lots of type II fibers, especially IIB, tends to produce meat with a low level of tenderness [15, 29]. This was also seen in Aceh cows. Both types of muscle in Aceh cows have type II muscle fibers which are quite high so that the impact on the level of meat tenderness is low. Type II muscle fibers are larger in size compared to type I fibers [30], which contributes to lean meat.

The connective tissue components found in skeletal muscle include collagen, proteoglycans, and cross-links [16]. The study results also prove that the collagen content is influenced by muscle type. Listrat et al. [16] proved that intramuscular connective tissue varies between muscle types and livestock types. Collagen is found more in the perimysium layer of both muscles than in the endomysium layer. The toughness of Aceh beef is also thought to be influenced by the amount and solubility of collagen; however, collagen analysis in this study was conducted qualitatively so that the percentage of dissolved and undissolved collagen was not certain. Joo et al. [31] stated that the amount and solubility of collagen are determining factors for meat tenderness. A large amount of total collagen in muscles indicates that the meat produced from these muscles is tighter [32]. The results of the correlation analysis show that total collagen, insoluble collagen, and cross-links have a significant negative correlation with meat tenderness, while dissolved collagen has a positive correlation with meat tenderness [15]. In future studies, it is very important to analyze each collagen component in Acehnese beef to determine the factors that most influence the tenderness of Aceh beef.

The results of the proximate analysis showed that the levels of protein, fat, water, and ash between the two muscle types of Aceh beef were not different, and this meant that the muscle type did not affect the nutritional value of the meat. The fat content of Aceh beef tends to be low with relatively high water content. Fat, protein, water, and ash content are influenced by cattle breed, genetics, livestock final care systems before slaughter, and meat handling [33–35]. The low-fat content in Aceh cattle is thought to be since Aceh cows are only fed grass without giving the concentrate. Cows that are fed only grass tend to produce meat with a low-fat content [36]. The low-fat content in Aceh cattle is also thought to be due to the nonoptimal slaughter age of Aceh cattle (nonoptimal maturity level). The maturity level of livestock largely determines the percentage of meat fat content [37]. Meat with high intramuscular fat content tends to have high-fat content and low water content, and vice versa [4]. Based on the results of the proximate analysis, it shows that the Aceh beef from both muscles has the same nutritional value, so that both can be choices for consumers.

The pH_24_ Aceh beef is in an acceptable pH range and is in the normal pH range for beef based on SNI 3932 of 2008, namely, 5.40–5.80. The results of the study prove that the pH of the meat is not only influenced by livestock breeds but also by the type of muscle that makes up the meat. LL muscles have a lower pH_24_ than ST muscles because LL muscles are thought to have higher glycolytic activity. In postmortem conditions, a high glycolytic activity can convert glycogen to lactic acid, so that the final pH of the meat becomes more acidic. In addition, a decrease in meat pH also occurs due to excess glycogen in muscles [38]. The final rearing system before the livestock is slaughtered largely determines the final pH of the meat. Livestock that are fed only grass will produce meat with a high pH [33]. However, this is not proven in this study. Genetics does not affect the pH of meat produced [33]; however, Ramos et al. [39] reported that the LL muscle of Brahman cattle (*Bos indicus*) tended to be resistant to lowering pH in postmortem conditions compared to Angus cattle (*Bos taurus*) and Brangus cattle (crosses of *Bos taurus* and *Bos indicus*). The LL Brahman cattle muscle is thought to be more resistant to cellular stress due to various factors during postmortem conditions. The pH value of meat has a positive correlation with water-binding capacity. Meat with low pH has a low water binding capacity so that a lot of water will come out of the meat [40]. The color of meat from the LL and ST muscles of Aceh beef did not differ significantly. The color of the meat is related to the pH and the binding capacity of the water. The higher the brightness level of the meat, the lower the pH value and the binding capacity of the meat water [35]. Aceh beef LL and ST muscles have the same fat color.

Intramuscular fat (marbling) is a determining factor for eating quality. The LL muscle has intramuscular fat, whereas the ST muscle has no intramuscular fat. These results prove that muscle type affects intramuscular fat levels. Intramuscular fat differs between muscle types even in the same breed of cattle [15, 28, 41]. The presence of intramuscular fat is also determined by the type of livestock [16]. Gálvez et al. [41] stated that muscles that are oxidative and have little physical activity have higher intramuscular fat than muscles that are glycolytic and have high physical activity. LL muscles are slow oxidoglycolytic (contain many types of IIA fibers) and include muscles that function to maintain livestock posture compared to ST muscles which are fast glycolytic and active in livestock movement [15]. The intramuscular fat content is also influenced by breed and gender, feed and weight, and age of slaughter [5]. Aceh cows are classified as cows with low intramuscular fat content, in contrast to exotic cows such as Wagyu cattle which are known to have high intramuscular fat. Aceh cows that are raised using the traditional system are only given feed in the form of forage without giving concentrate. These cows are raised with simple management without implementing a fattening program like the feedlot. Park et al. [5] stated that bulls fed only forage will produce meat with a lower intramuscular fat content and this increases with the age of the cattle. Aceh cows are slaughtered without paying attention to the proper age of slaughter so that the development of intramuscular fat is not optimal.

The WBSF value of Aceh beef obtained in this study is relatively high. The WBSF value shows the level of the tenderness of the meat [42]; thus, the Aceh beef originating from LL and ST muscles tends to have a low level of tenderness. The results of the study are also supported by previous studies [43]. Meat tenderness is influenced by several factors, including cattle breed, slaughter age, muscle protein degradation, and collagen content [15, 39, 44]) and the size of muscle fibers as shown in this study. Bressan et al. [33] proved that *Bos indicus* cattle have a higher WBSF value compared to *Bos taurus* cattle. Ramos et al. [39] stated that cows crossed between *Bos indicus* and *Bos taurus* have an adaptation mechanism of muscle cells to fight cell death in postmortem conditions. The high energy availability at the beginning of the postmortem and the delay in the autolysis process by calpain-1 resulted in a decrease in pH being inhibited and ultimately slowing the conversion process of muscle to meat. This adaptation mechanism is considered able to delay the meat tender process of *Bos taurus indicus*.

Cooking losses can be affected by livestock breeds [34]. The cooking losses and water holding capacity between the two types of Aceh beef muscle did not differ significantly. The high cooking loss is considered caused by the high water content in Aceh beef, especially free water, so that when the meat is cooked, a lot of free water is released. The relatively high percentage of cooking loss tends to be a result of the low water holding capacity of the meat. This can be seen from the percentage of water binding capacity in the two types of muscles of the Aceh cows. High water content but cannot be bound in the meat will cause a lot of water content to leak out when the meat is cooked. Water holding capacity has a negative correlation with the moisture content of meat [40].

The results of the qPCR analysis showed that the MSTN gene expressed in the two muscle types with relative quantification values was not different. This result is supported by Albrecht et al. [45]. This study does not show that muscle type affects myostatin gene expression. MSTN gene expression is relatively the same between LL and ST muscles, but the area of fibers between the two muscles is significantly different. Individual response or muscle adaptation response is thought to be different between LL and ST muscles so that the resulting phenotypes are also different even though they have relatively the same MSTN gene expression. The level of myostatin gene expression can be influenced by livestock breeds and heat stress [46]. The increase in MSTN gene expression is often followed by a decrease in the expression of structural genes in muscles such as myosin heavy chain (MHC) and desmin genes, which will result in loss of muscle mass [47]. Genetic factors influence MSTN gene expression and are breed-specific [48]. The MSTN gene has a close relationship with the growth rate and carcass characteristics so that this gene has the potential to be studied more intensively so that it can be implemented in the cattle fattening program so that good quantity and quality of meat can be obtained [49].

LL and ST muscles are the two types of muscle that make up meat that is often purchased by beef consumers in Indonesia. Cooking methods can affect the quality of meat desired by consumers, such as taste and tenderness [50]. The study conducted by Sofyan et al. [9] notes that most people in Aceh Province like Aceh beef because it tastes delicious and sweet, does not crumble easily and shrinks after cooking, and has a finer fiber compared to imported meat fiber. Therefore, the low level of the tenderness of Aceh beef obtained in this study is still suitable and following the type of cuisine of the people in Indonesia. Indonesia has several types of beef-based dishes that are processed using the wet cooking method and cooked for a long time, such as rendang and curry [9, 51]. The habit of cooking by boiling for a long time was influenced by the Indian culture which brought its influence to the cooking methods of people in Indonesia [52].

The characteristics of Indonesian cuisine are influenced by natural and cultural conditions. Indonesian cuisine can be classified based on the six major islands as Sumatera, Java, Sulawesi, Kalimantan, Bali, and Papua. Each region has different food cultural characteristics which are shaped by natural, historical, and cultural conditions. The food on the island of Sumatera (including Aceh Province) is heavily influenced by Middle Eastern and Indian cultures. The dishes have a very strong taste (spicy, sour, thick which comes from the heavy use of coconut milk in cooking dishes). The influence of India can mostly be seen in the dishes that are generally cooked by the people of Aceh which are made from beef, such as meat curry dishes that use lots of spices such as cloves and nutmeg as well as those used in Indian cuisine [52]. Curry is a type of dish made from beef or mutton that is often cooked by Aceh beef consumers [9] and is also one of the main types of cuisine in Aceh Province [53, 54]. Apart from curry, there are other types of dishes made from beef, including jerky, curry, kuwah beulangong, sie reuboh, and meat soup [53]. In the manufacturing process, all types of dishes undergo a relatively long boiling process [54]. Therefore, based on how to cook various main dishes of Aceh people, Aceh beef which is used as a raw material for making various types of culinary does not require a high level of tenderness.

Aceh cattle are the result of cross-breeding between *Bos javanicus* and *Bos indicus* [55]. Several studies have shown that *Bos indicus* have a lower level of meat tenderness compared to *Bos taurus* [33, 39], so that, genetically, Aceh cattle tend to have a low level of meat tenderness. Generally, Aceh cattle are kept by breeders for 6 months to 1 year before being sold to the animal market. The length of time for rearing also has an impact on the thickness of the meat produced. The level of the tenderness of Aceh beef according to the type of dish cooked by consumers is a form of food culture that underlies an identity of local Aceh people which must be maintained in the future as a cultural heritage as stated by Zeng et al. [56] and Guererro et al. [57].

## 5. Conclusion

Based on the muscle microstructure variable and the MSTN gene expression, it can be seen that the quality of Aceh beef is still relatively low when compared to the quality of imported meat from exotic cows because it has a low level of meat tenderness. The level of the tenderness of the meat is low because the meat is composed of large muscle fibers with a high percentage of type II muscle fibers. However, Aceh beef originating from people's farms is suitable for the cooking methods and types of food cooked by the community. Aceh beef has also a low-fat content so that it has the potential as a healthy food choice for consumers. The amino acid analysis is needed to complete the information on the quality of Aceh beef traditionally raised by breeders. Based on the study results, grass-feeding without concentrate can be maintained but the quality and quantity of grass can be improved. In addition, the study results can also add references to improve the quality standards of Indonesian local beef which are adapted to the sociocultural conditions (local wisdom) of the people in Indonesia.

## Figures and Tables

**Table 1:** The specific primer sequences of MSTN and *β*-actin used in gene expression analysis.

Gene	Primer sequences	PCR product (bp)
MSTN	Forward 5′-GAGAGATGCCAGCAGTGACG-3′	213
	Reverse 3′-GTCGCAGGAGTCTTGACAGG -5′	
*β*-Actin	Forward 5′-GGACTTCGAGCAGGAGATGG-3′	172
	Reverse 3′-GGTAGTTTCGTGAATGCCGC-5′	

**Table 2:** Area, diameter, and the number of muscle fibers (types I and II) in the longissimus lumborum (LL) and semitendinosus (ST) muscles of Aceh cows.

Variable	Muscle type		*p* value
	LL (mean ± SD) (*n* = 378)	ST (mean ± SD) (*n* = 351)	
Area (*μ*m^2^)	3 409.66 ± 1 072.27^a^	3 566.70 ± 1 077.20^b^	0.029
Diameter (*μ*m)	45.58 ± 7.95^a^	46.80 ± 7.07^a^	0.067
Muscle fiber (%)^*∗*^			
Type I	17.63 ± 13.96^a^	8.20 ± 3.20^a^	0.236
Type II	82.37 ± 13.96^a^	91.80 ± 3.20^a^	0.236

*n*: the number of muscle fibers measured; SD: standard deviation. Means in the same row with different superscripts differ significantly (*p* < 0.05). ^*∗*^Calculation of the number of types of muscle fibers is carried out in one field of view for each muscle sample.

**Table 3:** Distribution of collagen in the longissimus lumborum (LL) and semitendinosus (ST) muscles of Aceh cows.

Muscle type	Endomysium layer	Perimysium layer
LL	+	+∼++
ST	+	+∼+++

+: thin distribution; ++: moderate distribution; +++: thick distribution.

**Table 4:** The proximate value of Aceh beef.

Variables	Muscle		*p* value
	LL (mean ± SD) (*N* = 17)	ST (mean ± SD) (*N* = 16)	
Protein (%)	20.90 ± 1.27^a^	21.61 ± 1.33^a^	0.126
Fat (%)	2.69 ± 2.00^a^	2.16 ± 1.57^a^	0.403
Water (%)	73.89 ± 2.26^a^	73.25 ± 3.57^a^	0.505
Ash (%)	1.83 ± 0.54^a^	2.06 ± 0.43^a^	0.187

LL: longissimus lumborum; ST: semitendinosus; SD: standard deviation. Means in the same row with different superscripts differ significantly (*p* < 0.05).

**Table 5:** Physicochemical quality of Aceh beef.

Variables	Muscle		*p* value
	LL (mean ± SD) (*n* = 14)	ST (mean ± SD) (*n* = 14)	
pH_24_	5.38 ± 0.10^a^	5.59 ± 0.13^b^	0.000
Color (visual score)	3.00 ± 0.00^a^	2.36 ± 0.74^b^	0.003
Fat color (visual score)	3.00 ± 0.00^a^	3.00 ± 0.00^a^	-
Intramuscular fat (visual score)	1.29 ± 0.47^a^	0.00 ± 0.00^b^	0.000
WBSF (kg cm^−2^)	7.59 ± 1.36^a^	7.69 ± 1.26^a^	0.711
Decreased cooking (%)	47.95 ± 3.34^a^	46.16 ± 5.01^a^	0.376
Water holding capacity (%)	29.86 ± 2.80^a^	29.96 ± 1.50^a^	0.430

LL: longissimus lumborum; ST: semitendinosus; SD: standard deviation. Means in the same row with different superscripts differ significantly (*p* < 0.05).

**Table 6:** The correlation coefficient between the muscle microstructure and meat quality in the longissimus lumborum (LL) and semitendinosus (ST) muscles of Aceh cows.

Microstructure variable	mRNA MSTN	pH_24_	WBSF	Cooking losses	Water holding capacity	Fat	Water
*Longissimus lumborum (LL)*							
Area of muscle fibers	−0.94	−0.09	−0.82	−0.36	0.47	−0.46	−0.51
Type I muscle fibers	0.84	−0.64	0.16	−0.29	−0.97^*∗*^	0.96^*∗*^	0.94
Type II muscle fibers	−0.84	0.64	−0.16	0.29	0.97^*∗*^	−0.96^*∗*^	−0.94

*Semitendinosus (ST)*							
Area of muscle fibers	0.62	0.14	0.06	−0.73	0.02	−0.46	−0.51
Type I muscle fibers	−0.81	0.75	0.79	0.61	0.80	0.96^*∗*^	0.94
Type II muscle fibers	0.81	−0.75	−0.79	−0.61	−0.80	−0.96^*∗*^	−0.94

^*∗*^There is a correlation at a significance of 0.05.

## Data Availability

The data used to support the findings of this study are available from the corresponding author upon request by email, hamny@unsyiah.ac.id.
